# Effect of different storage conditions on the detection stability of vitamin K2 by liquid chromatography and tandem mass spectrometry

**DOI:** 10.1016/j.plabm.2026.e00558

**Published:** 2026-10-14

**Authors:** Chunyan Li, Shangqi Yin, Jiayi He, Xinqi Tang, Ruichen Liu, Xiaojun Li, Hailing Chen

**Affiliations:** a Beijing Jishuitan Hospital, Capital Medical University, Beijing, China; b Yanjing Medical College, Capital Medical University, Beijing, China; c Peking University Fourth School of Clinical Medicine, Peking University Health Science Center, Beijing, China; d Shanghai AB Sciex Analytical Instrument Trading Co., Ltd., Beijing, China; e Department of Osteoporosis, Beijing Jishuitan Hospital, Capital Medical University, Beijing, China

**Keywords:** Liquid chromatography-tandem mass spectrometry (LC-MS/MS), Vitamin K2 (VK2), Menaquinone-4 (MK-4), Menaquinone-7 (MK-7), Storage conditions, Sample stability, Recovery rate

## Abstract

**Objective:**

The influence of different storage conditions on the stability of vitamin K2 (VK2) was investigated to identify the optimal storage method to ensure the accuracy of Vitamin K2 detection by LC-MS/MS.

**Methods:**

A total of 100 serum samples collected from January to March 2025 at Beijing Jishuitan Hospital, Capital Medical University, were divided into four groups: light-shielded frozen, light-shielded room temperature, light-shielded refrigerated, and light-exposed room temperature for testing. The LC-MS/MS method was used to determine the levels of VK2 supplements tetramethoxybenzoquinone (MK-4) and heptamethoxybenzoquinone (MK-7), with low, medium, and high concentration groups selected respectively. The recovery rates of MK-4 and MK-7 between groups were calculated and compared.

**Results:**

The recovery rates of MK-4 under light-exclusion conditions for 1 day were: QCL: 94.10%, QCM: 110.94%, QCH: 106.99%. For MK-7 under light-exclusion conditions at room temperature for 1 day, the rates were: QCL: 88.55%, QCM: 89.96%, QCH: 78.84%. Under light-exposure conditions at room temperature for 7 days, the recovery rates of MK-4 decreased to: QCL: 19.38%, QCM:13.17%, QCH:18.32%. For MK-7 under light-exposure conditions at room temperature for 7 days, the rates dropped to:QCL:28.51%,QCM:24.43%,QCH:22.01%.

**Conclusion:**

This study demonstrates that storage conditions significantly influence the stability of VK2 determination by LC-MS/MS. Light exposure notably affects MK-7 detection stability, while storage temperature impacts MK-4 stability. For VK2 samples analyzed via LC-MS/MS, we recommend light-protected storage: 4-8°C refrigeration under lightfree conditions for short-term testing within 7 days, and-20°C freezing under light-free conditions for extended testing periods. These findings provide experimental evidence for optimizing storage protocols and quality control strategies in VK2 analysis using LC-MS/MS technology.

## Highlights

• First comparative study on light and temperature effects on VK2 stability, revealing MK-4 and MK-7 differences.
• 7-day light exposure at room temperature reduced MK-7 recovery to as low as 22%, highlighting light protection as critical.
• Optimal VK2 storage: 4–8°C refrigerated (short-term) and −20°C frozen (long-term), both light-protected.
• Provides evidence-based storage protocols and quality control strategies for LC-MS/MS analysis of VK2.
• Distinct temperature sensitivity for MK-4 vs. light sensitivity for MK-7 demonstrated in serum samples.

## Graphical abstract

**Figure:**
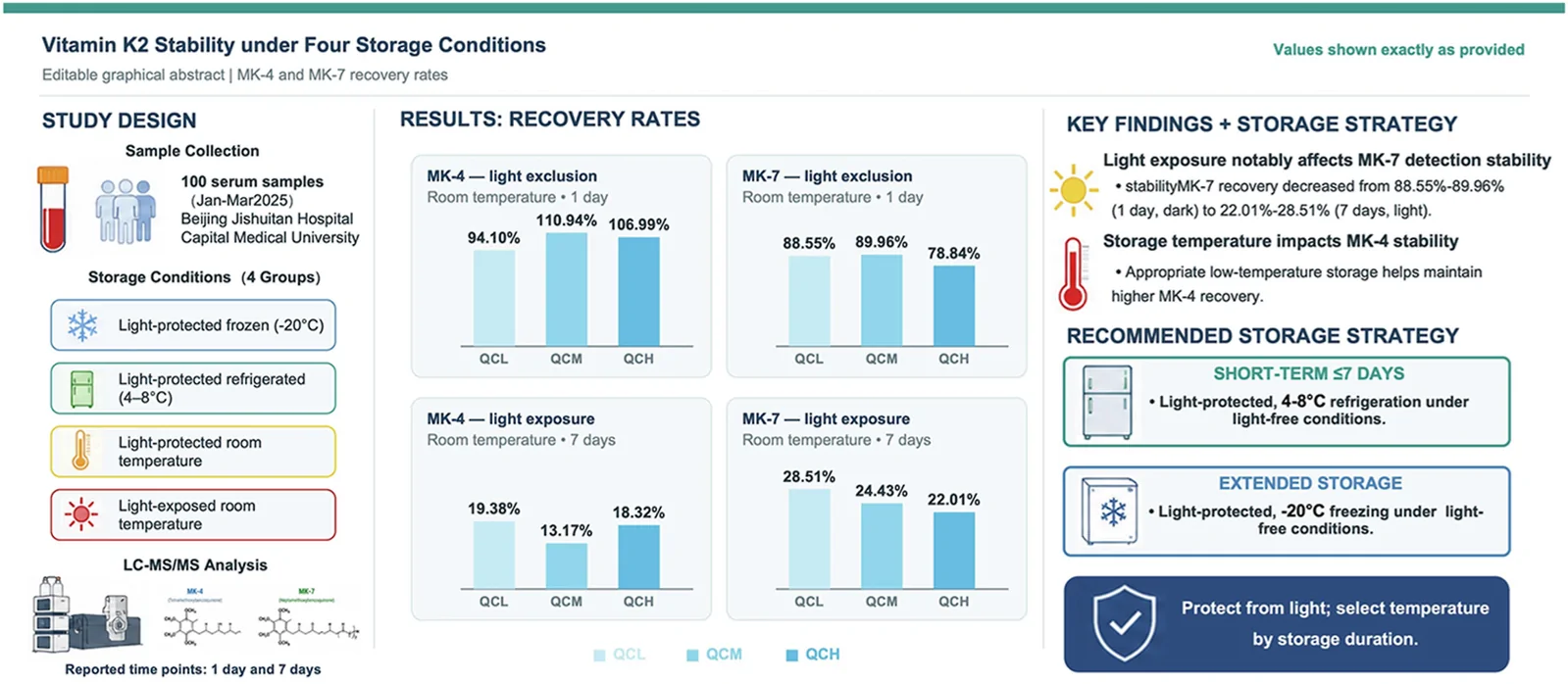

## 1 Introduction

Since the 1960s, mass spectrometric methods have been increasingly applied in clinical laboratories. For example, liquid chromatography–tandem mass spectrometry (LC–MS/MS) has been used to quantify small molecules such as vitamin K2 [1].

Vitamin K2 (VK2, menaquinones, MK-n) is a group of fat-soluble vitamins characterized by an isoprenoid side chain [2].It is widely present in fermented foods and animal-derived products, primarily synthesized by bacteria [3], [4], [5].As a plays critical roles in various physiological processes in humans. Among its most notable forms are menaquinone-4 (MK-4) and menaquinone-7 (MK-7): MK-4, mainly derived from animal-based foods such as eggs, meat, and liver, exhibits the highest biological activity among menaquinones; MK-7, predominantly found in fermented foods, demonstrates high bioavailability and an extended half-life approximately 72 h [6], [7], [8].VK2 contributes significantly to the regulation of bone metabolism and soft tissue calcification, particularly by facilitating the carboxylation of vitamin K-dependent proteins such as osteocalcin [9], [10], [11].Beyond its well-established role in coagulation, VK2 also exerts important physiological functions in bone metabolism, vascular health, energy metabolism, and inflammation regulation [12], [13]. However, the traditional immunoassay detection of Vitamin K2 was unreliable because of cross-reactivity.

Currently, liquid chromatography-tandem mass spectrometry (LC-MS/MS) has become the mainstream analytical method for determining vitamin K2 (particularly MK-4 and MK-7) in plasma as it provides more accurate measurement [14].However, due to its low basal concentration in blood and sensitivity to environmental factors such as light and heat, VK2 is prone to degradation during sample handling and storage, leading to deviations in measurement [15], [16].Therefore, systematically evaluating the impact of different storage conditions on VK2 stability and establishing standardized protocols for sample pretreatment and storage are of great significance for improving the accuracy and reliability of LC-MS/MS detection [17]. This study aims to provide a scientific basis and practical guidance for high-quality clinical VK2 detection by comparing the stability performance of VK2 under various storage conditions.

## 2 Materials and methods

### 2.1 Chemicals and reagents

Reference standards MK-4 and MK-7 were purchased from Sigma-Aldrich (St. Louis, MO, USA). Deuterated internal standards MK-4-d7 and MK-7-d7 were obtained from Toronto Research Chemicals (Canada). High-performance liquid chromatography-HPLC-grade solvents including methanol, acetonitrile, isopropanol, and isooctane, were acquired from Sigma-Aldrich (USA). Ultra-pure water (Milli-Q, 18.2 M cm) was supplied by Merck Millipore (Germany). Formic acid (HPLC grade) was sourced from Thermo Fisher Scientific (Waltham, MA, USA).

### 2.2 Instrument

An Agilent 1290 Infinity II ultra-high performance liquid chromatography system (Agilent Technologies, USA) coupled with an Agilent 6460 Triple Quadrupole mass spectrometer (Agilent Technologies, USA) was employed. The quantification of VK2 supplements MK-4 and MK-7 in plasma samples was performed using a Calibra CQS LC-MS/MS system (Calibra Precision Medical Laboratory Technology Co., Ltd., Beijing, China), equipped with Analyst 1.6.3 data acquisition software and MultiQuant quantitative analysis software. The ionization was conducted in positive ion mode via an atmospheric pressure chemical ionization (APCI) source. Multiple reaction monitoring (MRM) was used for mass spectrometric detection. Clot activator tubes (Vacuette, Greiner Bio-One, Essen, Germany) were used for sample collection.

### 2.3 LC–MS/MS conditions

Chromatographic separation was achieved using a Kinetex phenyl-hexyl column (C8, 2.5 μm, 2.1 × 100 mm) with a mobile phase comprising 0.1% formic acid in water (phase A) and 0.1% formic acid in methanol (phase B). Phase A was prepared by adding 1 mL of additive to 500 mL of a 50% aqueous methanol solution, while phase B was prepared by adding 1 mL of additive to 500 mL of methanol, both followed by 10-min ultrasonication [18]. Mass spectrometric detection was performed in multiple reaction monitoring (MRM) mode using electrospray ionization in positive ion mode (ESI+). Key ion source parameters included a capillary voltage of 1.5 kV, desolvation temperature of 600°C, desolvation gas flow rate of 1100 μL/h, and ion source temperature of 150°C. Detailed MRM transition parameters are summarized in Table 1.

**Table 1:** Multiple reaction monitoring mode ion pair parameters.

Compound	Precursor Ion (Q1, *m*/*z*)	Product Ion (Q3, *m*/*z*)	Dwell Time (s)	Declustering Potential (V)	Collision Energy (V)
MK-4	445.3	187.1	0.263	36	18
Menatetrenone-d7	452.4	194.2	0.263	38	22
MK-7	649.6	187.1	0.291	54	28
Menaquinone-13C6	655.5	193.1	0.4	52	28

Note: MK-4 is Menatetrenone; MK-7 is Menaquinone-7. Menatetrenone-d7(deuterium-labeled) and Menatetrenone-13C6(13Carbon-labeled): Stable isotope-labeled internal standards employed in LC-MS/MS bioanalysis for the precise quantification of native Menatetrenone.

### 2.4 Methods

Serum samples were collected from 50 osteoporosis patients and 50 healthy controls who underwent testing at Beijing Jishuitan Hospital, Capital Medical University, between January and March 2025. All samples were drawn into clot-activator tubes (Vacuette®, Greiner Bio-One GmbH, Essen, Germany). After centrifugation at 3726 × g for 10 min, the sera were separated and stored at −80°C. The study protocol was reviewed and approved by the hospital's Medical Ethics Committee. As shown in Table 2, all samples were divided into four parts and stored under four conditions: protected from light at −20°C, 2-8°C, and 20-25°C (room temperature), as well as exposed to light at 20-25°C. The concentrations of MK-4 and MK-7 were measured, and their recovery rates were calculated after 1, 2 and 7 days.

**Table 2:**
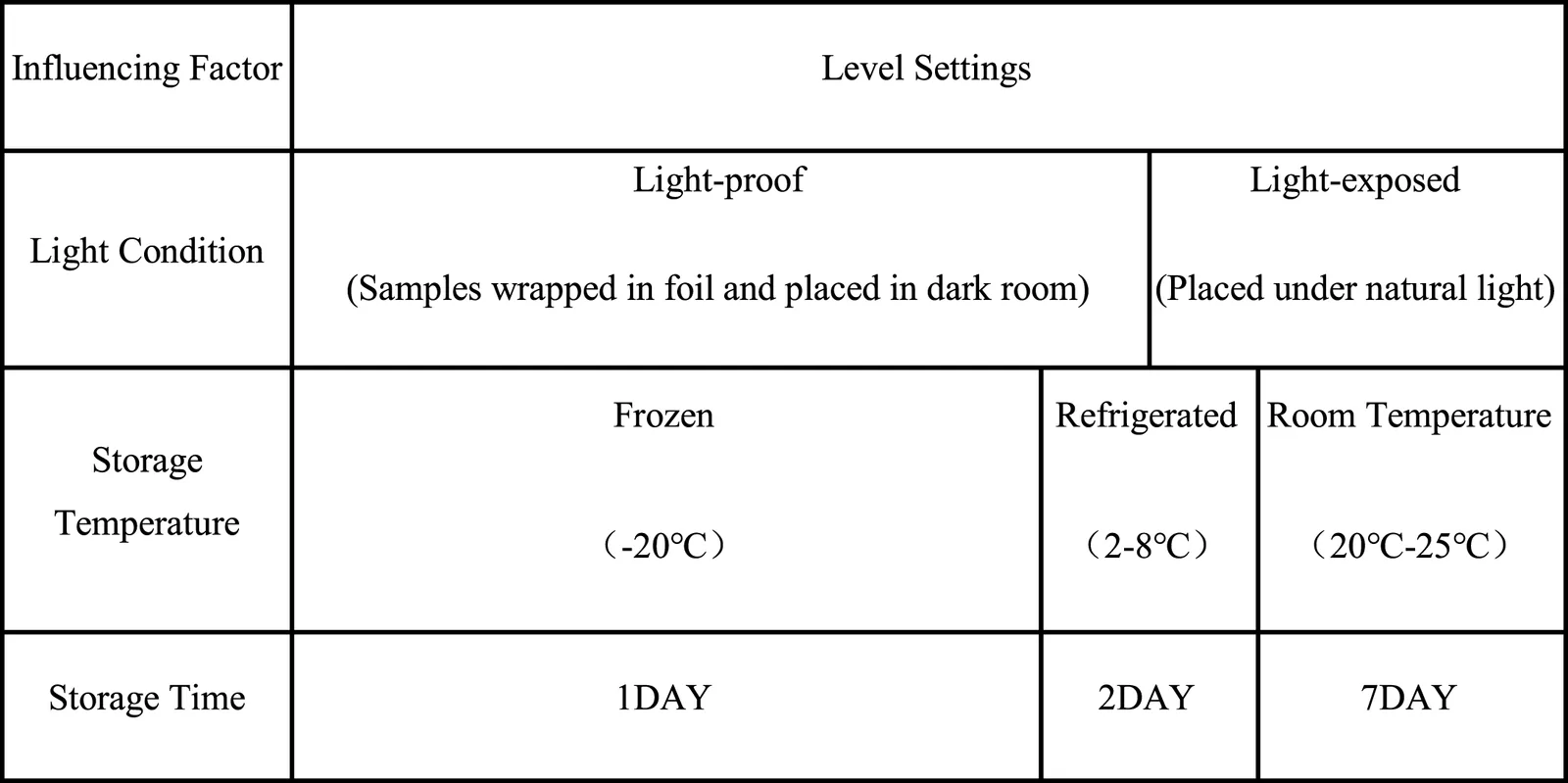
Experimental Condition Group Settings.

#### 2.4.1 Sample pretreatment

250uL of plasma was pipetted into a 2 mL polypropylene centrifuge tube. Then, 50 L of isotopic internal standard working solution and 1 mL of protein precipitant (acetonitrile with 1% formic acid) were added. The mixture was vortexed for 2 min. After centrifugation at 10,000 × g for 10 min (4°C), the supernatant was transferred to a new 2 mL glass centrifuge tube.

#### 2.4.2 Liquid-Liquid Extraction (LLE)

2 mL of isooctane (chromatographic grade) was added to the supernatant. The mixture was vortexed vigorously for 2 min and then centrifuged at 2150 × g for 10 min (20°C) to achieve clear separation of the organic (upper) and aqueous (lower) phases. The upper organic phase was transferred to a 5 mL glass tube using a glass pipette. The extraction was repeated once by adding another 2 mL of isooctane to the original aqueous phase, repeating the vortexing and centrifugation steps, and combining the organic phases. The combined organic phase was evaporated to dryness under a gentle nitrogen stream in a 35°C water bath. The residue was immediately reconstituted with 500 μL of 90% ethanol, vortexed for 2 min.

### 2.5 Criteria for assessing method stability

The recovery rate of VK2 is typically used to evaluate the accuracy of the detection method. For each analytical batch, five quality control (QC) samples at three different concentrations (low, medium, and high) are analyzed. This provides a relatively objective means to assess the stability of the LC-MS/MS method for VK2 detection. The acceptable recovery rate range for VK2 is generally considered to be between 80% and 120%, though it can be influenced by the specific detection method (e.g., HPLC, LC-MS), the sample pretreatment procedure, and experimental operational precision. In practice, if the recovery rate falls within this range, it indicates that the extraction, separation, and quantification of VK2 by the method are relatively accurate, and interference from the sample matrix (e.g., proteins, lipids in blood) is minimal. If the recovery rate deviates significantly from this range (e.g., below 70% or above 130%), it may suggest excessive loss during the pretreatment steps, interference within the detection method itself, or errors in the standard addition operation, necessitating investigation and optimization of the experimental procedure.

The recovery rate is calculated based on the known amount of standard added and the amount actually detected, using the following formula: Recovery Rate (%) = [(Amount Actually Detected - Native Amount in Sample)/Amount of Standard Added] × 100%

Detailed Explanation: Native Amount in Sample: The detected concentration of VK2 in the original blood sample without added standard. Amount of Standard Added: The known amount of VK2 standard deliberately added to the sample (calculated based on the added volume and concentration). Amount Actually Detected: The total amount of VK2 detected in the sample after the standard has been added.

### 2.6 Statistical methods

Statistical analysis was performed using SPSS software (version 26.0). Measurement data conforming to a normal distribution are expressed as the mean ± standard deviation (X ± s), and comparisons between two groups were conducted using the independent samples *t*-test. Data not conforming to a normal distribution are expressed as the median and interquartile range [M (P25, P75)], and comparisons were made using the non-parametric Mann-Whitney *U* test. A two-tailed p-value of less than 0.05 was considered statistically significant.

## 3 Result

### 3.1 Storage temperature and the stability of MK-4

As shown in Table 3, the recovery rates of MK-4 after 1 day of storage under protected from light and frozen conditions could reach QCL: 94.10%; QCM: 110.94%; QCH: 106.99%. These values were significantly higher than those observed after 1 day of storage under protected from light/refrigerated conditions and protected from light/room temperature conditions. Therefore, it is recommended to store samples at an appropriate temperature. Generally, samples can be stored short-term in a 4°C refrigerator, while long-term storage requires freezing at −20°C or −80°C.

**Table 3:** Recovery rates of MK-4 under different storage conditions.

		Group 1 - Light-proof Frozen	Group 2 - Light-proof Refrigerated	Group 3 - Light-proof Room Temperature	Group 4 - Light-exposed Room Temperature
1DAY Recovery Rate	QCL	94.1	92.2	63.7	61.62
	QCM	110.94	97.5	78.69	72.21
	QCH	106.99	103.6	83.42	78.23

2DAY Recovery Rate	QCL	106.15	99.7	43.04	39.65
	QCM	125.5	105	56.73	37.92
	QCH	104.52	91.83	46.32	36.09

7DAY Recovery Rate	QCL	90.35	71.8	22.51	19.38
	QCM	85.2	43.93	23.34	13.17
	QCH	93.93	74.29	23.67	18.32

### 3.2 Light conditions and the stability of MK-7

As shown in Table 3, The recovery rates of MK-7 after 1 day of storage under protected from light and room temperature conditions reached QCL: 88.55%; QCM: 89.96%; QCH: 78.84% (Table 3, Table 4). These values were significantly higher than those of the group exposed to light at room temperature for 1 day. Therefore, it is recommended that samples be stored protected from light. Prolonged exposure to high temperatures or strong light should be avoided to prevent the oxidation of VK2, which can lead to its degradation and loss, consequently affecting the recovery rate.

**Table 4:** Recovery rates of MK-7 under different storage conditions.

		Group 1 - Light-proof Frozen	Group 2 - Light-proof Refrigerated	Group 3 - Light-proof Room Temperature	Group 4 - Light-exposed Room Temperature
1DAY Recovery Rate	QCL	87.59	99.1	88.55	64.49
	QCM	99.11	95.2	89.96	62.22
	QCH	86.05	107.3	78.84	65.93

2DAY Recovery Rate	QCL	91.67	99.9	70.73	40.78
	QCM	87.89	101.2	71.43	44.48
	QCH	85.26	91.06	76.07	46.29

7DAY Recovery Rate	QCL	92.26	62.03	31.64	28.51
	QCM	85.47	61.44	23.22	24.43
	QCH	88.01	58.44	29.04	22.01

### 3.3 Storage duration: storage duration and the stability of both MK-4 and MK-7

The recovery rate of MK-4 after 7 days of storage under exposed to light and room temperature conditions was as low as QCL: 19.38%; QCM: 13.17%; QCH: 18.32%. Similarly, the recovery rate of MK-7 under the same conditions was as low as QCL: 28.51%; QCM: 24.43%; QCH: 22.01% (Table 3, Table 4). These recovery rates were significantly lower than those of samples stored for 2 days and 1 day, with the 1day storage group showing relatively higher recovery rates. This indicates that the degree of degradation of MK-4 and MK-7 is proportional to time; the longer the storage duration, the greater the degradation. Therefore, it is recommended that blood samples be processed as soon as possible after collection. If testing cannot be performed promptly, an appropriate storage method must be selected for sample preservation.

## 4 Discussion

This study systematically investigated the effects of different storage conditions on the stability of VK2 (represented by MK-4 and MK-7) quantified by LCMS/MS, providing an experimental basis for sample pretreatment and storage in actual testing procedures. The quinone ring and unsaturated side chain in the structure of VK2 make it highly sensitive to environmental factors such as light, heat, and oxygen, leading to potential degradation under non-standard storage conditions, which significantly compromises the accuracy of quantification [19], [20].By simulating common storage scenarios in actual testing workflows, this study identified temperature, time, and light exposure as critical factors affecting VK2 stability, and accordingly proposed targeted recommendations for sample storage.

There are many mechanisms underlying the effect of storage conditions on VK2 Stability. Temperature can influence the chemical stability of both MK-4 and MK-7, particularly MK-4 [7].High temperatures may trigger chemical reactions such as hydrolysis or oxidation of MK-4 and MK-7, thereby altering their chemical structures and concentrations and affecting the detection results [21]. In the LC-MS/MS analysis of MK-4 and MK-7, variations in column temperature can also lead to abnormal retention times; for instance, ambient temperature fluctuations exceeding 3°C can cause pressure variations, consequently affecting retention times. Unsuitable temperatures may impair the separation efficiency of MK-4 and MK-7 from other compounds, compromising the accuracy of quantitative analysis [18]. High temperatures accelerate redox reactions involving the quinone ring of MK-4 and MK-7, while also promoting the cleavage of double bonds in the isoprenoid side chain units, leading to a decrease in content [22]. Conversely, low temperatures can slow degradation by reducing molecular motion rates and inhibiting the activity of oxidative enzymes. Therefore, low-temperature storage is essential for ensuring consistency and accuracy in LC-MS/MS detection of MK-4 and MK-7 [23]. What's more, specific spectral radiation, particularly ultraviolet and visible light, may induce photochemical reactions in certain samples or the stationary phase, altering their properties and subsequently affecting chromatographic behavior. MK-4 and MK-7 are light-sensitive compounds, with MK-7 being especially sensitive [7]. Light exposure can cause structural changes, generating photodegradation products that may interfere with the detection of MK-4 and MK-7, leading to inaccurate results. Therefore, light-protected storage is a necessary condition to ensure experimental consistency and accuracy in the LC-MS/MS detection of MK-4 and MK-7. Thus, for biological samples such as serum, if testing is to be completed within 24 h, short-term storage at 4°C protected from light is acceptable; if the storage period is within 7 days, samples should be stored at −20°C under light-protected and frozen conditions, and aliquoting in advance is advised to avoid repeated freeze-thaw cycles; for storage durations exceeding 7 days, −80°C deep-freezing with light protection is recommended [24].Furthermore, during the testing process, samples should not be kept at room temperature for more than 60 min after removal from the freezer, and the use of brown injection vials is recommended to effectively prevent potential light-induced degradation during analysis.

## 5 Limitation

This study also has several limitations. First, it focused solely on two homologs, MK-4 and MK-7, without including other structural analogs (such as MK-9), thus failing to comprehensively reflect the stability profile of the entire VK2 family [25], [26].Second, the investigation was limited to serum as the biological matrix and did not extend to other matrices such as tissues or cells, where complex lipid backgrounds may exert additional effects on VK2 stability [27], [28].Furthermore, while the study primarily addressed the impact of pre-analytical storage conditions, it did not thoroughly explore other potential influencing factors during sample processing, such as the type of anticoagulant or centrifugation conditions. Future research should expand to include stability assessments of other VK2 homologs and investigate optimal storage conditions across different biological matrices. Additionally, the incorporation of stable isotope-labeled internal standards (e.g., ^13^C-labeled MK-7) could more accurately correct for analyte loss during extraction and storage, thereby enhancing the method's resistance to matrix interference. It would also be valuable to further investigate the protective effects of antioxidants or stabilizers on VK2 during sample storage, with the aim of developing more effective sample preservation protocols.

## 6 Conclusion

This study demonstrates that storage conditions are the primary determinant of vitamin K_2_ (K2) stability in LC-MS/MS analysis. Experimental data reveal that light exposure is a critical variable affecting the stability of MK-7 measurements, while storage temperature governs the preservation of MK-4. Accordingly, a tiered storage protocol is recommended: samples intended for analysis within 7 days should be refrigerated at 4–8°C in light-protected conditions; for long-term storage, freezing at −20°C in the dark is required. These findings provide direct experimental support for standardizing storage procedures and enhancing quality control in LC-MS/MS-based VK2 analysis.

## CRediT authorship contribution statement

**Chunyan Li:** Conceptualization, Methodology, Formal analysis, Investigation, Writing – original draft. **Shangqi Yin:** Investigation, Data curation, Writing – review & editing, Visualization. **Jiayi He:** Investigation, Methodology, Validation, Writing – review & editing. **Xinqi Tang:** Investigation, Data curation, Writing – review & editing. **Ruichen Liu:** Investigation, Formal analysis, Software, Writing – review & editing. **Xiaojun Li:** Conceptualization, Resources, Supervision, Project administration, Funding acquisition, Writing – review & editing. **Hailing Chen:** Resources, Supervision, Methodology, Writing – review & editing.

## Ethics approval and consent to participate

This study was approved by the Ethics Committee of Beijing Jishuitan Hospital (Approval No.202203-05).

## Availability of data and materials

All the data generated or analyzed during this study are included in this published article and its supplementary information files.

## Funding sources

Supported by Beijing Natural Science Foundation (No. 7262062) Beijing JST Research Funding (YGQ-202605).

## Declaration of competing interest

The authors declare that they have no known competing financial interests or personal relationships that could have appeared to influence the work reported in this paper.

## Data Availability

The data that has been used is confidential.
